# 

**Published:** 1895-04-20

**Authors:** 


					AFBIL L'UTIT, 1895.
WITH EXTRA NURSING SUPPLEMENT.
Per Annnm, 10s. 6d? post free.
Uonthly Farts, 6d.; post tree. 8d.
Ditto per Annnm, 8s. 8d? post free.
? uIascow Western Infirmabj - -NORfOLhAua Norwich hospijam
No. 447. vol. xvm. with EXTRA NURSING SUPPLEMENT. Saturday, April 20tit, 1895.

				

